# *Bacillus* Probiotics as Alternatives to In-feed Antibiotics and Its Influence on Growth, Serum Chemistry, Antioxidant Status, Intestinal Histomorphology, and Lesion Scores in Disease-Challenged Broiler Chickens

**DOI:** 10.3389/fvets.2022.876725

**Published:** 2022-04-28

**Authors:** Ifeanyi Princewill Ogbuewu, Monnye Mabelebele, Nthabiseng Amenda Sebola, Christian Mbajiorgu

**Affiliations:** ^1^Department of Agriculture and Animal Health, University of South Africa, Florida, South Africa; ^2^Department of Animal Science and Technology, Federal University of Technology, Owerri, Nigeria

**Keywords:** broiler chickens, *Bacillus*, antibiotic alternatives, growth, health makers

## Abstract

In commercial poultry production, chickens are reared under intensive conditions, which may allow infections to spread quickly. Antibiotics are used at sub-therapeutic doses in livestock and poultry feed to prevent diseases and improve productivity. However, restrictions on the use of antibiotics at sub-therapeutic concentrations in livestock feed due to growing concerns of antimicrobial resistance (AMR), together with antibiotic residues in meat and eggs has prompted poultry researchers and feed producers to look for viable alternatives. Thus, there is increasing interest in developing natural alternatives to in-feed antibiotics to improve chicken productivity and health. Probiotics, specifically from the genus *Bacillus* have proven to be effective due to their spore-forming capabilities. Furthermore, their ability to withstand heat during feed processing and be stored for a long time without losing viability as well as their potential to function in the acidic medium of the chicken gut, provide them with several advantages over conventional probiotics. Several studies regarding the antimicrobial and antioxidant activities of *Bacillus* probiotics and their positive impact in chicken nutrition have been documented. Therefore, the present review shields light on the positive effect of *Bacillus* probiotics as alternatives to in-feed antibiotics on growth performance, serum chemistry, antioxidant status, intestinal histomorphology and lesion scores of disease-challenged broiler chickens and the mechanisms by which they exert their actions. It is concluded that *Bacillus* probiotics supplementation improve growth, health and productive indices of disease-challenged broiler chickens and can be a good alternative to in-feed antibiotics. However, more studies are required on the effect of *Bacillus* probiotics supplementation in broiler chickens to maximize productivity and achieve the ultimate goal of stopping the usage of antibiotics at sub-therapeutic doses in broiler chicken feed to enhance performance.

## Introduction

Demand for poultry source foods is increasing, particularly in developing countries (1), and this is driven primarily by population growth (2, 3). To keep pace with the growing request for poultry products, farmers now use antibiotics at minute doses to reduce the incidence of enteric pathogens, improve feed conversion ratio (FCR) and body weight gain (BWG) in broiler chickens (4). Despite the significant economic benefit of AGPs in large-scale broiler production, their continued use in animal feed has been heavily criticized because of growing concerns of AMR and the occurrence of multi-drug resistant bacteria. In view of this, several countries have banned the use of antibiotics at sub-therapeutic levels in feed for food animal production (5).

The ban on the use of antibiotics at sub-therapeutic doses in livestock feed resulted in economic losses because of reduced feed efficiency, decreased feed intake, BWG, higher morbidity and mortality of broiler chickens as a result of high prevalence of pathogens (6, 7). In addition, in-feed antibiotics increased the incidence of food-borne illness in humans (8). To maneuver the limitation linked to the usage of in-feed antibiotics in animal agriculture, poultry nutritionists and farmers have proposed the use of probiotics found to have no residual effect on animal products or result in antimicrobial resistance (9). A probiotic is viable or inactivated micro-organisms that exert beneficial effects to the host when administered in an adequate amount. *Lactobacillus spp*. is one of the common probiotics used in broiler chicken feed because they are considered safe, naturally present in the gut and with demonstrable health-promoting effects (10, 11). Other conventional probiotics with known beneficial impact in chickens include *Saccharomyces spp*., *Aspergillus spp*., Enterococcus *spp*., and *Bifidobacteria* (12–15). The use of these probiotics is still limited as challenges have been noted during feed preparations, whereby these organisms are not able to withstand the high temperature of the feed pelletisation process. Furthermore, reduced shelf life and limited survival in the gut has to date, contributed to the poor adoption by the farmers into routine broiler production process (16) The limitations of conventional chicken probiotics during industrial production processes have been documented in the literature (17).

The use of *Bacillus* probiotics in broiler chicken feed is gaining attention since it has features that address the drawbacks related to Lactobacillus based probiotics. *Bacillus* organisms are gram-positive bacterium with the ability to form spores. The ability of *Bacillus* organisms to form spores ensures that they remain stable and viable during feed manufacturing processes, storage, and movement through the gastrointestinal tract (17–19) thus implying that *Bacillus* products are suitable for adoption in the poultry industry. Besides, evidence also exists that *Bacillus* strains were produced at high efficiency (20) and hence, one of the key advantages of using *Bacilli* as feed probiotics is their capability to resist the changing conditions in the gastrointestinal tract of chickens (17, 21). Importantly, as suggested by Ramlucken et al. (17) *Bacillus* spores can retain about 90% of their viability during the probiotic harvesting process and have a 5-year shelf life potential.

In another study, Aly et al. (22) prepared three probiotic supplemented diets, with diet 1 containing a mixture of 0.5 × 10^7^ *L. acidophilus*/g feed and 0.5 × 10^7^ *B. subtilis*/g feed, and diets 2 and 3 containing 1 × 10^7^ L. acidophilus/g feed and 1 × 10^7^ *B. subtilis*/g feed, respectively, and stored for 4 weeks at 4°C and 25°C. After, 4 weeks of storage at 4°C and 25°C, the authors discovered that diets 1 and 2 had significantly more viable cells than diet 3 after 4 weeks of storage at 4°C and 25°C. Broilers chickens challenged with *C. perfringens* fed a diet supplemented with *B. coagulans* experienced less intestinal damage and consumed more feed than those fed a diet supplemented with *L. fermentum* (23). In agreement with the present findings, Brzoska et al. (24) and Olnood et al. (25) found that dietary *Lactococcus lactis* and *Lactobacillus* spp. supplementation did not improve feed intake, FCR and BWG (24, 25), all of which are important parameters for probiotic acceptance in the broiler chicken industry. The poor performance of chickens on conventional probiotics supplementation (24, 25) compared to probiotic *Bacillus* could be partly attributed to their low survivability in the harsh conditions of the gastrointestinal tract (26, 27).

*Bacillus* probiotics used as probiotics in humans and animals include *B. coagulans, B. cereus, B. subtilis* and *B. licheniformis* (28, 29). Several studies have found that feeding *Bacillus* spp. to chickens enhances their growth and productivity (30–32). However, the impact of *Bacillus* probiotics supplementation on the performance indices of disease-challenged broiler chickens were inconclusive due to differences in strain of *Bacillus* probiotics used, dosage, the severity of infection, age of chickens, and rearing environment (7, 33).

Thus, the purpose of this review is to highlight the possible mechanisms of action of *Bacillus* probiotics and the influence of dietary *Bacillus* probiotics supplementation on growth parameters, blood metabolites, intestinal histomorphological indices and lesion scores in disease-challenged broiler chickens. In addition, the antioxidant and antimicrobial properties of *Bacillus* spp. in disease-challenged broiler chickens were discussed. We also discussed several conflicting research findings, reasons for these differences and offer recommendations on the potential of *Bacillus* spp. to replace to in-feed antibiotics in disease-challenged broiler chickens.

## Mechanisms of Action

The probable mechanisms by which *Bacillus* spp. limit the proliferation of pathogens include competition for adhesion sites, production of organic acids leading to a reduction in gut pH, maintenance of normal gut microbiota *via* competitive exclusion (CE) and antagonisms, production of antimicrobial compounds, improvement in oxidative stability, modulation of immune system, improvement in digestive enzyme activity and competition for nutrients (21, 34). The inhibitory effect may be achieved through one or a combination of these actions. Probiotics improve the immune system by inducing the production antimicrobial compounds, and raising the concentrations of secretory immunoglobulin A (35). Upon consumption, *Bacillus* spp. alters the intestinal environment and produces a variety of digestive enzymes which improves digestibility and nutrient absorption in poultry (36, 37). The mechanism of competition for binding sites on the intestinal mucosa is mediated by glycocalyx, a layer that protects the intestinal epithelial from mechanical damage and prevents pathogen colonization, thus protecting the host from infections (38). CE is one method for controlling enteric pathogens and zoonotic agents in chickens (8). Competition for adhesion sites on the intestinal mucosa is influenced by the pH of the digesta (21). Low pH promotes the growth of acidophilic bacteria such as *Bacillus* probiotics, which inhibit the proliferation of enteric pathogens (*Salmonella* spp., *Clostridium perfringens* and *Escherichia coli*) using the CE technique. *Bacillus* spp. has also been reported to produce bacteriocins which are toxic to enteric pathogens (39–41).

## Sources of Bacillus Probiotics and Criteria For Its Desirable Probiotic Properties

Currently, the *Bacillus* probiotics used in livestock production are isolated from the intestine, food, soil and pond. They are widely found in the dust, water, air, and soil (21). Several companies have successfully commercialized *Bacillus* probiotic products as detailed by Ramlucken et al. (17) in their recent review. The most common *Bacillus* probiotics used in livestock and poultry research is *B. subtilis* or its strains. *Bacillus* probiotics are identified based on standard morphological, biochemical, physiological tests, 16S rRNA gene sequencing or multilocus sequence analysis. To exert its positive influence on the host, probiotic bacteria should be able to survive, bind to the intestinal mucosa, maintain good viability, utilize the nutrients and substrates found in the diet, and remain non-pathogenic and non-toxic. *Bacillus* probiotic comes in two types: vegetative and spore. The vegetative type is destroyed by gastric acid and bile salts, but spores thrive in both conditions (42). Studies have shown that *Bacillus* probiotic produces different kinds of AMPs including bacteriocins, glycopeptides, lipopeptides, and cyclic peptides (39, 43). Bacteriocins are toxic to pathogens and are one the criteria for selecting a probiotic strain.

## Antioxidant Status

*Bacillus* probiotics respond to oxidative stress by upregulation of catalases and thioredoxins (44). Abudabos et al. (30) reported that Salmonella-challenged broilers treated with *B. subtilis*-based probiotics had decreased serum concentrations of total antioxidant capacity (TAC), whereas those treated with avilamycin had increased serum concentrations of TAC. The significantly increased serum levels of TAC in Salmonella-challenged broilers fed antibiotic-based diet is consistent with Kabploy et al. (45), who linked the avilamycin's anti-oxidative activity to its scavenging effect on hydroxyl radicals. On the other hand, Rajput et al. (46) recorded higher serum TAC concentrations in *S. enterica* challenged birds fed diets containing mixtures of *S. boulardii* and *B. subtilis* B10. Available information also revealed that probiotic bacteria can boost antioxidant status of broiler chickens (47). Free radicals are produced in chickens during an inflammatory process, and superoxide dismutase (SOD) is a zinc-based antioxidant enzyme that helps to degrade free radicals (48). Carillon et al. (49) found that chickens infected with 1 × 10^4^ *S*. *enteritidis* cfu/bird had higher SOD concentration at 3 and 10 days of age than challenged broilers administered 10^4^ spore/g *B. subtilis*. The authors hypothesized that the elevated SOD activity in *S. enteritidis*-challenged broilers could be attributed to the ability of chickens to counteract the oxidative damage caused by severe intestinal injury induced by *S. enteritidis* toxins, since SOD plays an important part in mitigating oxidative damage (49).

## Antimicrobial Property

There are studies concerning the inhibitory influence of *Bacillus* probiotics on gram-positive bacteria (39, 50, 51). *B. subtilis* supplementation at 10^4^ and 10^6^ spores/g reduces the proliferation of Salmonella (gram-negative bacteria) in the crop, proventriculus and intestinal compartments of broiler chickens infected with *S. enteritidis* at 10^4^ cfu (48). However, they did not detect Salmonella in the intestine when *B. subtilis* was given at a higher dose (10^6^ spores/g), implying that *B. subtilis* has dose-related antibacterial effects in broiler chickens. Tactacan et al. (52) observed a similar pattern in disease-challenged broiler chickens that received 10^4^ and 10^6^ cfu/g *B. subtilis* (QST 713) for 28 days. Although the detailed mechanisms of antibacterial properties of *Bacillus* probiotics are unknown, however, they may have achieved this *via* maintenance of gut microbial ecology through CE and antagonism, production of AMPs, enhancement of digestive enzyme activity, stimulation of immune system and improvement in absorptive efficiency of the small intestine (33, 53, 54).

The addition of *B. subtilis* to chicken feed decreased *C. perfringens* (36, 55, 56), Enterobacteriaceae (36) and Campylobacter (57) count in the excreta. *Bacillus* spp. supplementation has been reported to reduce *C. perfringens* proliferation in the intestine (53). In a similar study, Deepak et al. (58) noticed that *C. perfringens* counts (log_10_ cfu/g of jejunal content) were significantly reduced in infected broilers administered *B. subtilis*. In agreement, Jayaraman et al. (56) and Lin et al. (59) discovered that dietary *B. subtilis* supplementation reduces *C. perfringens* proliferation in the gastrointestinal tracts of broiler chickens. Likewise, Liu et al. (60) observed a decline in microbial diversity of *C. perfringens* infected broilers fed *B. subtilis* PB6 supplemented diets. Arif et al. (61) observed that *Bacillus* probiotics supplementation increased the population of *Bacillus* spp. on day 21 and 35, while decreasing the proliferation of *C. perfringens, Salmonella* spp. and *E. coli* in the jejunum and ileum of broiler chickens reared under a low-level of biosecurity measures. The exact mechanisms that lead to the inhibitory activity of *B. subtilis* against enteric pathogens are not clear. However, according to Cladera-Olivera et al. (43) and Baindara et al. (39), *Bacillus* probiotics produce bacteriocins that are active against other pathogens.

## Growth Performance

Influence of dietary *Bacillus* probiotics on feed intake, FCR and BWG of disease-challenged broiler chickens has been documented (Table 1). Abudabos et al. (30) found that administration of *B. subtilis* (2 × 10^7^ cfu/g) and avilamycin (0.2 g/kg) to infected broiler chickens improved growth performance indices when compared with the infected control broilers, implying that *B. subtilis* could replace antibiotics in poultry nutrition. A similar finding has been reported by Roy et al. (62) in heat stressed broiler chickens fed *B. subtilis* (0.5 g/kg feed), 2.2% lincomycin (0.15 g/kg feed) and their blend (0.5 and 0.15 g/kg feed, respectively). The improvement in FCR could be linked to the capability of *B. subtilis* to increase production of digestive enzymes, which help in the breakdown of feed into smaller fractions that are easily absorbed by the chickens. Adhikari et al. (48) reported higher BWG and lower FCR in *S. typhimurium* and *C. perfringens* challenged broilers fed *Bacillus* probiotics in comparison with antibiotics. The beneficial effect of *Bacillus* probiotics on the gut microflora could lead to improved BWG and FCR (21). Furthermore, Abudabos et al. (63) reported higher BWG in Salmonella-challenged broilers fed 2 × 10^7^ cfu/g *B. subtilis* in contrast to those fed diets supplemented with avilamycin at 0.2 g/kg and infected control, suggesting that probiotics may be a viable alternative to antibiotics. The authors also showed that infected broilers fed a basal diet without feed additives performed poorly in terms of BWG, FCR and villi morphology compared to non-challenged control broilers, confirming the negative impact of Salmonella infection in chicken growth and productivity (64). Abudabos et al. (63) recorded superior BWG in broilers fed *B. subtilis* compared to the broilers fed *B. licheniformis*, and the observed contradiction could be ascribed to differences in *Bacillus* strain used and dosage (33).

**Table 1:** Influence of *Bacillus* probiotics on broiler performance.

***Bacillus* strain**	**Broiler strain**	**Dosage (cfu/g)**	**Duration of study (days)**	**Pathogen** **challenged**	**Response variables**	**Authors**
*Bacillus cereus* var. toyoii	Ross	1 ×10^6^ spores/g	47	*S. enteritidis*	*Bacillus cereus* var. toyoii significantly improved FCR and BWG in challenged groups compared to the infected control.	(65)
*B. licheniformis* (DSM17236)	Cobb	8 ×10^5^ 8 ×10^6^ 8 ×10^7^	28	*C. perfringens*	Broilers fed diets having three inclusion levels of *B. licheniformis* and virginiamycin (15 g/ton) had significantly lower FCR, and higher BWG than the NE-challenged control.	(66)
*B. subtilis (DSM17299)*	Cobb	8 ×10^5^	42	*Salmonella* Heidelberg	Broilers on *B. subtilis* had higher BWG than the challenged control	(67)
*B. subtilis* PB6 (ATCC-PTA 6737)	Cobb	5.0 ×10^7^	35	*C. perfringens*	Challenged broiler chickens fed *B. subtilis* PB6 supplemented feed 5 ×10^7^ cfu/g had significantly better body weight gain and feed efficiency than infected control and non-infected control birds.	(56)
*B. subtilis (*Avicorr™)	Ross	1.5 ×10^5^	28	Eimeria infected litter	Birds in *B. subtilis*-treated group had better WG than the controls	(68)
*B. subtilis* (ATCC PTA- 6737)	Ross 308	2.0 ×10^7^	14	*S. typhimurium*	Higher (*p* > 0.05) WG and lower FCR were observed in the *B. subtilis* and avilamycin (0.2 g/kg) groups when compared to the challenged control group.	(30)
*B. licheniformis* H2	-	1.0 ×10^6^	22	*C. perfringens*	*B. licheniformis* improved BWG in *C. perfringens* challenged broiler chickens.	(69)
*B. subtilis* GalliPro-DSM 17299		1.2 ×10^6^	42	*C. perfringens*	NE-challenged broilers had depressed BWG while, infected broilers fed *B. subtilis* had significantly higher BWG. However, feed intake and FCR were not significantly influenced.	(58)
*Bacillus* DFMs	Ross 708	0.80 cfu/g *Bacillus* A. and 0.82 cfu/g *Bacillus* L	42	*Eimeria species* and *C. perfringens*	*Bacillus* DFMs supplementation increased BWG and reduced FCR and mortality in subclinical challenged broilers.	(70)
*B. licheniformis*	Avian	1.2 ×10^6^ cfu/g spore count	35	*C. perfringens*	Challenged broilers fed *B. licheniformis* and bacitracin methylene disalicylate (2 g/kg) had improved BWG and FCR than challenged control broilers.	(59)
*B. subtilis B21* *B. licheniformis B26*	Arbor Acre	2 ×10^9^	42	*C. perfringens*	Challenged broilers offered *Bacillus probiotics* and enramycin (5 mg/kg) recorded higher BWG and lower FCR than unchallenged control broilers.	(71)
*B. subtilis* *(ATCC PTA-* *6737)* *B. licheniformis*	Ross 308	2 ×10^7^ 2 ×10^6^	nr	*Salmonella* spp.	*Bacillus* probiotics supplementation increased feed intake and BWG in diseased challenged broilers.	(63)
*B. subtilis* PB6	Ross 308	0.5 g/kg	35	*C. perfringens*	NE challenged broilers fed *B. subtilis* additive had better FCR post-NE period (28–35 d) than NE challenged broilers offered diet without *B. subtilis* additive. In addition, non-challenged birds fed *B. subtilis* had the highest BWG followed by non-challenged birds fed basal diet only, while the lowest BWG were recorded in NE challenged birds without *B. subtilis*.	(72)
*B. amyloliquefaciens* LFB112	Ross 308	5 ×10^5^	39		Broilers fed *B. amyloliquefaciens* had heavier BWG and reduced FCR than birds fed *B. subtilis*, control (basal diet only) and antibiotics.	(73)
*B. subtilis* PB6	Arbor Acres	4 ×10^4^ 6 ×10^4^	26	*C. perfringens*	Dietary *Bacillus* probiotics increased feed intake, FCR and BWG in challenged broiler chickens The challenged groups administered *B. subtilis* PB6 compete favorably with the group administered with 10 mg/kg enramycin.	(60)

Low pH inhibits the growth and multiplication of pathogenic microbes (*S. typhimurium* and *C. perfringens*), while promoting the proliferation of beneficial microbes (33, 48, 74). It also facilitates the production and release of digestive enzymes such as pepsin, gastrin and cholecystokinin, all of which aid in digestion and nutrient utilization (33, 75). It is reported that *B. subtilis* PB6 improves feed intake, FCR and BWG in Necrotic enteritis (NE)-challenged broilers (72). Lee et al. (76) reported that *Bacillus* probiotics based DFMs ameliorated *Eimeria maxima* (EM)-induced reduction in BWG and intestinal lesions in broilers in comparison with EM infected control broilers. The growth-enhancing effect of *Bacillus* probiotics might be ascribed to it inhibitory effect against some enteric pathogens invasion in broilers (55, 77). This observation was consistent with Jayaraman et al. (56), who noticed an improvement in growth performance of *C. perfringens* infected broiler chickens fed *B. subtilis* PB6 additive. Addition of 1.0 kg *B. licheniformis*/metric ton (MT) of feed in broiler chickens reared for 35 days under a low-level of biosecurity measures significantly enhanced BWG and FCR compared to those fed basal diet with no additives and basal diet plus *Bacillus subtilis* at 1.0 kg/metric ton (MT) feed and 4% flavomycin at 0.3 kg/MT feed (61). Dietary *Bacillus* probiotic has been found to positively influence growth and productivity of disease-challenged broilers (64, 66–69), and may serve as an alternative to in-feed antibiotics in broiler feed.

Recently, Ahmat et al. (73) assessed the impact of *B. amyloliquefaciens* LFB112 and *B. subtilis* CICC 20179 on performance of broiler chickens and observed that broilers fed *B. amyloliquefaciens* at 5 × 10^5^ cfu/g had better BWG, FCR, carcass yield and cut-part weights (thigh and breast muscle) than broilers treated with *B. subtilis* (5 × 10^5^ cfu/g) and antibiotic (150 mg of aureomycin/kg). Based on their findings, supplementation of *B. subtilis* and aureomycin to the level of 5 × 10^5^ cfu/g and 150 mg/kg feed, respectively in broiler chickens did not enhance growth performance, suggesting that *B. subtilis* and aureomycin may not have reached the threshold required to improve growth and productivity in broilers. Abdominal fat is the most common way animal accumulate fat, and is positively correlated with total fat. Increased abdominal fat content connotes inefficient use of energy in the feed. It is evident from the findings of Ahmat et al. (73) that abdominal fat weights were significantly lower in birds offered diets supplemented with *B. amyloliquefaciens* (5 × 10^5^ cfu/g) and antibiotic (150 mg of aureomycin/kg) compared to control birds. This finding is in harmony with other authors (62, 78, 79), who found significantly lower abdominal fat weights in chickens fed *Bacillus* probiotics feed additives. The reduced abdominal fat pad weight could be due to a decrease in lipid content as probiotics have been reported to reduce the serum lipid content (63). The potential of *Bacillus* probiotics to limit the activity of fatty acid synthase in chicken liver could explain the significant reduction in abdominal fat pad.

## Intestinal Histomorphological Indices and Lesion Scores

Intestinal histomorphology is used as an important index for intestinal health and infection recovery. Studies have shown that broilers challenged with enteric pathogens had disorganized intestinal tight junctions, resulting in an increase in intestinal permeability (80, 81). A recent feeding study by Abudabos et al. (63) found deteriorated villi health in disease-challenged broiler chickens; however, treatment with *Bacillus* probiotics and antibiotics mitigated the negative effect. On the same hand, Hernandez-Patlan et al. (82) found that feeding *Bacillus* probiotic (1.0 × 10^6^ spores/g) to necrotic enteritis (NE) infected birds alleviated its adverse consequences. Similarly, Aljumaah et al. (72) found that 0.5 g/kg *B. subtilis* PB6 lowers intestinal lesion scores in NE challenged-broilers suggesting enhanced recovery. This reduction in intestinal lesion scores may be attributed to *Bacillus* probiotics' ability to eliminate enteric pathogens through competitive exclusion and antagonisms (55, 70).

Jayaraman et al. (56) investigated the impact of *B. subtilis* administration on the micro-anatomy of broiler chickens challenged with *C. perfringens*-induced NE and NE-challenged control birds had thickened mucosa, hemorrhages, intestinal lesions, and ballooning of the intestine. However, there were no signs of gross pathological changes in the intestines of NE-challenged broilers fed *B. subtilis* PB6 (5 × 10^7^ cfu/g), indicating that inclusion of *B. subtilis* PB6 in chicken feed reduced intestinal *C. perfringens* counts and improved gut integrity. Jayaraman et al. (56) also reported distorted and damaged villi in *C. perfringens* challenged broiler chickens, implying loss of gut integrity. These findings also supported others who reported improved intestinal health in disease-challenged broilers (63, 83, 84) and healthy broiler chickens (31, 85, 86) fed *Bacillus* probiotics in their diets. The improved intestinal health in both healthy and infected broilers could be due to a decrease in the population of enteric pathogenic microbes, leading to increased digestion and utilization. These results corroborated Samanya and Yamauchi (87), who reported improved intestinal villus measurements in healthy chicken fed dried *B. subtilis*.

Deepak et al. (58) found severe histopathological changes in the jejunal villi of NE-challenged broiler chickens. However, the severity of the histopathological changes was reduced in infected broilers treated with *B. subtilis* compared to infected control birds. They also observed that the NE-challenged broilers administered *B. subtilis* had increased villi length compared to challenged control broiler chickens. In a similar study, Lee et al. (76) noticed significantly reduced intestinal lesion scores in EM infected broiler chickens fed diets treated with *B. subtilis* strains (15AP4 and Bs27) compared to infected control chickens. These findings are consistent with previous research (88–90). A recent investigation by Konieczka et al. (91) showed improved gut morpho-structural indices and structure in *C. perfringens* infected broilers fed multistrain *Bacillus* probiotic in their feed when compared to those fed single strain *Bacillus* probiotic, implying that the multistrain *Bacillus* probiotic was more effective than the single strain *Bacillus* probiotic in improving gut histomorphological indices in diseased broiler chickens.

*C. perfringens* produces several harmful substances after colonization of the gut (92), and this affects the tight junction and their components by increasing gut permeability, resulting in compromised gut integrity and function (81). Administration of antibiotics (enramycin) and *B. subtilis* (4.0–6.0 × 10^4^ cfu/g feed) restored gut integrity, and reduced intestinal lesion score in Arbor Acre broilers challenged with 10^8^ cfu/mL *C. perfringens* (60). Arif et al. (61) also found that inclusion of *B. licheniformis* at 1.0 kg/MT of feed in broiler chickens reared under a low-level of biosecurity measures had slightly better intestinal lesion score than groups fed diets with no additives, *Bacillus subtilis* PB6 (1.0 kg/MT feed) and 4% flavomycin (0.3 kg/MT feed). This agrees with Wu et al. (54), who found increased villus height (VH) and VH: crypt depth (CD) ratio in jejunum and reduced gut lesion scores in the small intestine in NE infected broilers fed a diet treated with *B. coagulans*. The damaged intestinal wall in NE-infected broilers could be one of the possible explanations for the poor growth performance of broilers infected with *C. perfringens*. Researchers have demonstrated that *B. subtilis* PB6 and enramycin can reduce lesion scores and enhance gut health in *C. perfringens* challenged broilers (71, 93). Enramycin kills enteric bacteria by inhibiting cell wall formation, reducing the harm they can cause to the body (93).

## Serum Biochemical Characteristics

Blood indices are used in the nutritional assessment to determine the quality of test feedstuffs/additives and to reflect the physiological state of the animal (94). Abudabos et al. (63) discovered that dietary *B. licheniformis* (2 × 10^6^ cfu/g) and *B. subtilis* (2 × 10^7^ cfu/g) significantly increased serum cholesterol, glucose, total protein and globulin levels in Salmonella-challenged broiler chickens. This is supported by the earlier findings of Abudabos et al. (84), who reported an improvement in plasma total protein and glucose levels in Salmonella-infected broilers fed diets having supplemental levels of *B. subtilis, S. boulardii* and oregano. The improvement in serum protein level could be ascribed to the ability of *Bacillus* probiotics to improve dietary protein digestion and utilization by increasing the absorptive efficiency of the small intestine (51, 95, 96). Also, Abudabos et al. (30) demonstrated that the inclusion of *B. subtilis* and antibiotics in the chicken diet improved serum biochemical values in Salmonella-challenged broilers. These findings were in converse with Abudabos et al. (63), who evaluated the impact of *B. subtilis* and avilamycin/kg feed on serum biochemical characteristics of Salmonella-challenged broilers and noticed that supplementation of *B. subtilis* and avilamycin/kg feed in broiler feed had no adverse influence on plasma cholesterol, glucose, total protein and globulin values. Abudabos et al. (83) stated that the addition of *B. subtilis* in the diets of Salmonella infected broilers during the starter phase did not affect the content of serum glucose and protein. They also reported that *B. amyloliquefaciens* supplementation influenced blood metabolites in broiler chickens during the starter phase. The reasons for the discrepancy in serum biochemical values in broilers fed *Bacillus* probiotics is unknown; however, the difference may be related to differences in strains of *Bacillus* probiotics and concentrations, as well as the severity of infection.

Serum immunoglobulins (Ig), especially those generated from the b cells are used as indicators of humoral immunological state in poultry because of their role in immune regulation and resistance to several diseases (97, 98). Serum Ig levels in broilers were affected by *Bacillus* probiotics as Ahmat et al. (73) observed increased serum IgA and IgG in healthy broilers fed aureomycin at 150 mg/kg, and *Bacillus* probiotic (*B. amyloliquefaciens* and *B. subtilis*) and their metabolites at the level of 5.0 × 10^5^ cfu/g in their diet. These results indicate that *Bacillus* probiotics and their metabolites that increased serum immunoglobulin concentrations will have more beneficial impacts on growth performance and broiler chickens' ability to combat diseases. This observation supports Luan et al. (99) and Wang et al. (100), who reported increased serum IgA, IgM and IgG in chickens fed *Bacillus* probiotics in their ration. This result supported Abudabos et al. (63) who found increased plasma globulin protein levels in Salmonella-challenged broiler chickens fed two strains of *Bacillus* probiotics and their metabolites. Rajput et al. (101) discovered that adding 1.0 × 10^5^ cfu/g *B. subtilis* B10 to chicken feed increased the amount of IgA-positive cells and concentration of cytokine secretory IgA in the small intestine.

## Dosage, Presentation Forms and Route Of Administration

Different effective doses of *Bacillus* probiotics in broilers have been reported (60, 63, 66, 71). However, the efficacy may vary from one study to the other due to differences in *Bacillus* probiotics composition, dosage, duration of supplementation and strain used as well as chicken's age and health status (21, 33). The effective dose of *Bacillus* probiotics in broilers ranges from 1.0 × 10^6^ to 8.0 × 10^7^ cfu/g feed (33). The most common form and route for *Bacillus* probiotics is powder administered *via* feed (33, 71). The powder form is preferred by end users as it has advantages such as uniformity, stability, longer shelf life, ease of packaging and transportation, and easy incorporation into current feed manufacturing processes (102–104).

## Limitations and Strengths

Some of the limitations of this study are: (1) In this review, we investigated the impact of *Bacillus* spp. on growth performance and health indices of disease-challenged broiler chickens and may not apply to other animal species; (ii) The variations in diet composition, degree of infection, species of *Bacillus* probiotics, dosage and duration of *Bacillus* probiotics supplementation and season may have effect on the reliability of the results. The differences in some of serum chemistry parameters in this review may depend on the age and sex of the chickens. In addition, differences in assay types and methods of analysis used by different studies may be a limitation. Despite constraints to their adoption, there is progressive increase in *Bacillus*-based probiotic studies used in poultry production. However, the results of this review have made an important contribution to knowledge on how *Bacillus* probiotics supplementation improves growth performance and health indices in disease-challenged broiler chickens.

## Conclusion

This review indicated that *Bacillus* probiotics currently used in broiler chicken industry are isolated from the gut, food, soil and pond. This study also revealed that *B. subtilis, B. coagulans* and *B. licheniformis* were the most common *Bacillus species* used in broiler chicken nutrition. In addition, *Bacillus* probiotics had a positive effect on body weight gain and feed conversion ratio. It also improved blood characteristics and intestinal histomorphological indices of disease-challenged broiler chickens and may replace in-feed antibiotics in broiler chicken production. These findings also support the proposition that *Bacillus species* can assist chickens to fight enteric pathogens. *Bacillus* probiotics may have achieved these beneficial effects through one or a blend of the following mechanisms of action: (1) decreasing pathogen proliferation in the gastrointestinal tracts *via* competitive exclusion and antagonism, (2) production of organic acids leading to a decline in gut pH, (3) production and release of antimicrobial compounds, (4) improvement in oxidative stability and immune systems, (5) modifications in gut metabolic processes to support the production and release of digestive enzymes, and (6) direct nutritional effects or enhancing feed consumption, digestibility and nutrient uptake. However, as with other probiotics, the potential of *Bacillus* probiotics in broiler chicken nutrition has not been fully realized, possibly because their effect is dependent on a variety of factors such as *Bacillus* strain, species, dose level, age of chicken among others. Thus, further research is required using meta-analysis and quadratic optimization model to determine the optimum dose level of *Bacillus* probiotics that enhance broiler chicken performance before *Bacillus* probiotics could be considered as a substitute for in-feed antibiotics. In addition, the use of transcriptomics technologies to understand the mechanisms by which of action of *Bacillus* probiotics improve health and productivity of disease-challenged broiler chickens is recommended as such information is lacking in the literature.

## Author Contributions

MM and IO: conceptualization. IO: writing—original draft preparation. NS, MM, and CM: review and editing. CM and MM: visualization. All authors have read and agreed to the published version of the manuscript.

## Conflict of Interest

The authors declare that the research was conducted in the absence of any commercial or financial relationships that could be construed as a potential conflict of interest.

## Publisher's Note

All claims expressed in this article are solely those of the authors and do not necessarily represent those of their affiliated organizations, or those of the publisher, the editors and the reviewers. Any product that may be evaluated in this article, or claim that may be made by its manufacturer, is not guaranteed or endorsed by the publisher.

## References

[1] ILRI. Options for the livestock sector in developing and emerging economies to 2030 and beyond. Meat: the future series. Geneva, Switzerland: World Economic Forum (2019).

[2] Zhou Y Staatz J. Projected demand and supply for various foods in West Africa: implications for investments and food policy. Food Policy. (2016) 61:198–212. 10.1016/j.foodpol.2016.04.002

[3] Desiere S Hung Y Verbeke W D'Haese M. Assessing current and future meat and fish consumption in Sub-Sahara Africa: learnings from FAO food balance sheets and LSMS household survey data. Glob Food Sec. (2018) 16:116–26. 10.1016/j.gfs.2017.12.004

[4] Engberg RM Hedemann MS Leser TD Jensen BB. Effect of zinc bacitracin and salinomycin on intestinal microflora and performance of broilers. Poult Sci. (2000) 79:1311–9. 10.1093/ps/79.9.1311 11020077

[5] FAO/WHO. Salmonella and Campylobacter in Chicken Meat: Meeting Report. Microbiological Risk Assessment Series 19. (2009). p. 1–42.

[6] Van Immerseel FV Rood JI Moore RJ Titball RW. Rethinking our understanding of the pathogenesis of necrotic enteritis in chickens. Trends in Microbiol. (2009) 17:32–6. 10.1016/j.tim.2008.09.005 18977143

[7] Ahiwe EU Dos Santos TT Graham H Iji PA. Can probiotic or prebiotic yeast (*Saccharomyces cerevisiae*) serve as alternatives to in-feed antibiotics for healthy or disease-challenged broiler chickens?: a review. J Appl Poult Res. (2021) 30:100164. 10.1016/j.japr.2021.100164

[8] Hao H Cheng G Iqbal Z Ai X Hussain HI Huang L. Benefits and risks of antimicrobial use in food-producing animals. Front Microbiol. (2014) 5:288. 10.3389/fmicb.2014.00288 24971079 PMC4054498

[9] Cheng G Hao H Xie S Wang X Dai M Huang L. Antibiotic alternatives: the substitution of antibiotics in animal husbandry? Front Microbiol. (2014) 5:69–83. 10.3389/fmicb.2014.00217 24860564 PMC4026712

[10] Kabir S. The role of probiotics in the poultry industry. Int J Mol Sci. (2009) 10:3531–46. 10.3390/ijms10083531 20111681 PMC2812824

[11] Patterson J Burkholder K. Application of prebiotics and probiotics in poultry production. Poult Sci. (2003) 82:627–31. 10.1093/ps/82.4.627 12710484

[12] Kim S Yu D Lee S Park S Ryu K Lee D. Effects of feeding Aspergillus oryzae ferments on performance, intestinal microfloua, blood serum components and environmental factors in broiler. Korean J Poult Sci. (2003) 30:151–9

[13] Reddy GV Panda N Anjaneyulu Y Reddy A. Khadar Vali. R Effect of yeast culture on the performance of broilers Indian J Anim Nut. (2005) 22:170–2

[14] Jung S Houde R Baurhoo B Zhao X Lee B. Effects of galacto-oligosaccharides and a Bifidobacteria lactis-based probiotic strain on the growth performance and fecal microflora of broiler chickens. Poult Sci. (2008) 87:1694–9. 10.3382/ps.2007-00489 18753434

[15] Franz CM Huch M Abriouel H Holzapfel W Gálvez A. Enterococci as probiotics and their implications in food safety. Int J Food Microbiol. (2011) 151:125–40. 10.1016/j.ijfoodmicro.2011.08.014 21962867

[16] Mattila-Sandholm T Myllärinen P Crittenden R Mogensen G Fondén R Saarela M. Technological challenges for future probiotic foods. Int Dairy J. (2002) 12:173–82. 10.1016/S0958-6946(01)00099-1

[17] Ramlucken U Lalloo R Roets Y Moonsamy G Jansen van Rensburg C Thantsha MS. Advantages of Bacillus-based probiotics in poultry production. Livest Sci. (2020) 241:104215. 10.1016/j.livsci.2020.104215

[18] Clavel T Carlin F Lairon D Nguyen-The C Schmitt P. Survival of *Bacillus cereus* spores and vegetative cells in acid media simulating human stomach. J Appl Microbiol. (2004) 97:214–9. 10.1111/j.1365-2672.2004.02292.x 15186458

[19] Bernardeau M Lehtinen M Forssten S Nurminen P. Importance of the gastrointestinal life cycle of *Bacillus* for probiotic functionality. J Food Sci Tech. (2017) 54:2570–84. 10.1007/s13197-017-2688-3 28740315 PMC5502041

[20] Ramlucken U Ramchuran SO Moonsamy G Jansen van Rensburg C Thantsha MS Lalloo R. Production and stability of a multi-strain Bacillus based probiotic product for commercial use in poultry. Biotechnol Rep. (2002) 29:e00575. 10.1016/j.btre.2020.e00575 33659192 PMC7890156

[21] Mingmongkolchai S Panbangred W. Bacillus probiotics: an alternative to antibiotics for livestock production. J Appld Microbiol. (2018) 124:1334–46. 10.1111/jam.13690 29316021

[22] Aly SM Ahmed YA Ghareeb AA Mohamed MF. Studies on *Bacillus subtilis* and *Lactobacillus acidophilus*, as potential probiotics, on the immune response and resistance of *Tilapia nilotica* (*Oreochromis niloticus*) to challenge infections. Fish and Shellfish Immunol. (2008) 25:128–36. 10.1016/j.fsi.2008.03.013 18450477

[23] Xia Y Zhang Y Xu J Guo S Ding B. Effects of *Lactobacillus* fermentum and *Bacillus coagulans* on the growth performance and gut health of broilers challenged with *Clostridium perfringens*. China Anim Husbandry Vet Med. (2019) 46:2927–36

[24] Brzoska F Sliwiński B Stecka K. Effect of Lactococcus lactis vs. *Lactobacillus* Spp. bacteria on chicken body weight, mortality, feed conversion and carcass quality. Ann Anim Sci. (2012) 12:549–59. 10.2478/v10220-012-0046-y

[25] Olnood CG Beski SS Choct M Iji PA. Novel probiotics: their effects on growth performance, gut development, microbial community and activity of broiler chickens. Anim Nutr. (2015) 1:184–91. 10.1016/j.aninu.2015.07.003 29767136 PMC5945945

[26] Taheri H Moravej H Tabandeh F Zaghari M Shivazad M. Screening of lactic acid bacteria toward their selection as a source of chicken probiotic. Poult Sci. (2009) 88:1586–93. 10.3382/ps.2009-00041 19590072

[27] Shokryazdan P Kalavathy R Sieo C Alitheen N Liang J Jahromi M. Isolation and characterization of Lactobacillus strains as potential probiotics for chickens. Pertanika J Trop Agric Sci. (2014) 37:141–57. 10.1155/2014/927268 25105147 PMC4106073

[28] Cutting SM. Bacillus probiotics. Food Microbiol. (2011) 28:214–20. 10.1016/j.fm.2010.03.007 21315976

[29] Fijan S. Microorganisms with claimed probiotic properties: an overview of recent literature. Int J Environ Res Public Health. (2014) 11:4745–67. 10.3390/ijerph110504745 24859749 PMC4053917

[30] Abudabos AM Alyemni AH Dafalla YM Khan RU. Effect of organic acid blend and Bacillus subtilis alone or in combination on growth traits, blood biochemical and antioxidant status in broilers exposed to Salmonella typhimurium challenge during the starter phase. J Appld Anim Res. (2017) 45:538–42. 10.1080/09712119.2016.1219665

[31] Bai K Feng C Jiang L Zhang L Zhang J Zhang L. Dietary effects of Bacillus subtilis fmbj on growth performance, small intestinal morphology, and its antioxidant capacity of broilers. Poult Sci. (2018) 97:2312–21. 10.3382/ps/pey116 29660060

[32] Dong Y Li R Liu Y Ma L Zha J Qiao X. Benefit of dietary supplementation with *Bacillus subtilis* BYS2 on growth performance, immune response, and disease resistance of broilers. Probio and Antimicrobial Prot. (2020) 12:1385–97. 10.1007/s12602-020-09643-w 32128666

[33] Ar'Quette G Gay CG Lillehoj HS. Bacillus spp. as direct fed microbial antibiotic alternatives to enhance growth, immunity, and gut health in poultry. Avian Path. (2018) 47:339–51. 10.1080/03079457.2018.1464117 29635926

[34] Khan RU Naz S. Application of probiotics in poultry production. World Poult Sci J. (2013) 69:621–32. 10.1017/S0043933913000627

[35] Xu X Huang Q Mao Y Cui Z Li Y Huang Y. Immunomodulatory effects of Bacillus subtilis (natto) B4 spores on murine macrophages. Microbiol Immunol. (2012) 56:817–24. 10.1111/j.1348-0421.2012.00508.x 22957751

[36] Jeong JS Kim IH. Effect of *Bacillus subtilis* C-3102 spores as a probiotic feed supplement on growth performance, noxious gas emission, and intestinal microflora in broilers. Poult Sci. (2014) 93:3097–103. 10.3382/ps.2014-04086 25260523

[37] Latorre JD Hernandez-Velasco X Wolfenden RE Vicente JL Wolfenden AD Menconi A. Evaluation and selection of *Bacillus* species based on enzyme production, antimicrobial activity, and biofilm synthesis as direct-fed microbial candidates for poultry. Front Vet Sci. (2016) 3:95. 10.3389/fvets.2016.00095 27812526 PMC5071321

[38] Soerjadi A Rufner R Snoeyenbos G Weinack OM. Adherence of Salmonellae and native gut microflora to the gastrointestinal mucosa of chicks. Avian Dis. (1982) 26:576–84. 10.2307/1589904 6756371

[39] Baindara P Mandal SM Chawla N Singh PK Pinnaka AK Korpole S. Characterization of two antimicrobial peptides produced by a halotolerant Bacillus subtilis strain SKDU4 isolated from a rhizosphere soil sample. AMB Express. (2013) 3:2. 10.1186/2191-0855-3-2 23289832 PMC3549917

[40] Khochamit N Siripornadulsil S Sukon P Siripornadulsil W. Antibacterial activity and genotypic–phenotypic characteristics of bacteriocin producing Bacillus subtilis KKU213: potential as a probiotic strain. Microbiol Res. (2015) 170:36–50. 10.1016/j.micres.2014.09.004 25440998

[41] Al-Khalaifah HS. Benefits of probiotics and/or prebiotics for antibiotic-reduced poultry. Poult Sci. (2018) 97:3807–15. 10.3382/ps/pey160 30165527

[42] Barbosa TM Serra CR La Ragione RM Woodward MJ Henriques AO. Screening for *Bacillus* isolates in the broiler gastrointestinal tract. Appl Environ Microbiol. (2005) 71:968–78. 10.1128/AEM.71.2.968-978.2005 15691955 PMC546680

[43] Cladera-Olivera F Caron GR Brandelli A. Bacteriocin-like substance production by *Bacillus licheniformis* strain P40. Lett Appl Microbiol. (2004) 38:251–6. 10.1111/j.1472-765X.2004.01478.x 15214721

[44] Mols M Abee M. Primary and secondary oxidative stress in Bacillus. Environtal Microbiol. (2011) 13:1387–94. 10.1111/j.1462-2920.2011.02433.x 21352461

[45] Kabploy K Bunyapraphatsara N Morales NP Paraksa N. Effect of antibiotic growth promoters on anti-oxidative and anti-inflammatory activities in broiler chickens. Thai J Vet Med. (2016) 46:89–95.

[46] Rajput IR Li YL Xu X Huang Y Zhi WC Yu DY. Supplementary effects of Saccharomyces boulardii and Bacillus subtilis B10 on digestive enzyme activities, antioxidation capacity and blood homeostasis in broiler. Int J Agric Biol. (2013) 15:231–7.

[47] Capcarova M Weis J Hrncar C Kolesarova A Pal G. Effect of *Lactobacillus fermentum* and *Enterococus faecium* strains on intestinal milieu, antioxidant status and body weight of broiler chickens. J Anim Physiol Anim Nutr. (2010) 94:215–24. 10.1111/j.1439-0396.2010.01010.x 20626505

[48] Adhikari B Hernandez-Patlan D Solis-Cruz B Kwon YM Arreguin MA Latorre JD. Evaluation of the antimicrobial and anti-inflammatory properties of *Bacillus*-DFM (NorumTM) in broiler chickens infected with *Salmonella enteritidis*. Frontiers in Vet Sci. (2019) 6:282. 10.3389/fvets.2019.00282 31508436 PMC6718558

[49] Carillon J Rouanet JM Cristol JP Brion R. Superoxide dismutase administration, a potential therapy against oxidative stress related diseases: several routes of supplementation and proposal of an original mechanism of action. Pharm Res. (2013) 30:2718–28. 10.1007/s11095-013-1113-5 23793992

[50] Yilmaz M Soran H Beyatli Y. Antimicrobial activities of some Bacillus spp. strains isolated from the soil. Microbiol Res. (2006) 161:127–31. 10.1016/j.micres.2005.07.001 16427515

[51] Kadaikunnan S Rejiniemon T Khaled JM Alharbi NS Mothana R. *In vitro*xs antibacterial, antifungal, antioxidant and functional properties of Bacillus amyloliquefaciens. Ann Clin Microbiol Antimicrob. (2015) 14:9. 10.1186/s12941-015-0069-1 25858278 PMC4342198

[52] Tactacan G Schmidt J Miille M Jimenez DA. *Bacillus subtilis* (QST 713) spore-based probiotic for necrotic enteritis control in broiler chickens. J Appl Poult Res. (2013) 22:825–31. 10.3382/japr.2013-00730

[53] Latorre JD Hernandez-Velasco X Kuttappan VA Wolfenden RE Vicente JL Wolfenden AD. Selection of Bacillus spp. for cellulase and xylanase production as direct-fed microbials to reduce digesta viscosity and Clostridium perfringens proliferation using an in vitro digestive model in different poultry diets. Front Vet Sci. (2015) 2:25. 10.3389/fvets.2015.00025 26664954 PMC4672186

[54] Wu Y Shao Y Song B Zhen W Wang Z Guo Y. Effects of *Bacillus* coagulans supplementation on the growth performance and gut health of broiler chickens with Clostridium perfringens-induced necrotic enteritis. J Anim Sci Biotechnol. (2018) 9:9. 10.1186/s40104-017-0220-2 29416856 PMC5784659

[55] Teo AYL Tan HM. Inhibition of Clostridium perfringens by a novel strain of Bacillus subtilis isolated from the gastrointestinal tracts of healthy chickens. Appl Environ Microbiol. (2005) 71:4185–90. 10.1128/AEM.71.8.4185-4190.2005 16085801 PMC1183296

[56] Jayaraman S Thangavel G Kurian H Mani R Mukkalil R Chirakkal H. Bacillus subtilis PB6 improves intestinal health of broiler chickens challenged with clostridium perfringens-induced necrotic enteritis. Poult Sci. (2013) 92:370–4. 10.3382/ps.2012-02528 23300303

[57] Guyard-Nicodeme M Keita A Quesne S Amelot M Poezevara T Le Berre B. Efficacy of feed additives against Campylobacter in live broilers during the entire rearing period. Poult Sci. (2016) 95:298–305. 10.3382/ps/pev303 26706356

[58] Deepak K Mukund K Mayura G Archana P Swati H Amit Y. Efficacy of *Bacillus subtilis* (GalliPro) supplementation in *Clostridium perfringens* challenged necrotic enteritis of broiler chicken. Indian J Anim Res. (2018) 52:619–22. 10.18805/ijar.B-3253

[59] Lin E Cheng Y Hsiao FS Proskura WS Dybus A Yu Y. Optimization of solid-state fermentation conditions of Bacillus licheniformis and its effects on Clostridium perfringens-induced necrotic enteritis in broilers. Revi Brasileira Zootec. (2019) 48:e20170298. 10.1590/rbz4820170298

[60] Liu Y Zhang S Luo Z Liu D. Supplemental *Bacillus subtilis* PB6 improves growth performance and gut health in broilers challenged with *Clostridium perfringens*. J Immunol Res. (2021) 2021:2549541. 10.1155/2021/2549541 34746321 PMC8566084

[61] Arif M Akteruzzaman M Tuhin-Al-Ferdous Islam SKS Das BC Siddique MP. Dietary supplementation of Bacillus-based probiotics on the growth performance, gut morphology, intestinal microbiota and immune response in low biosecurity broiler chickens. Vet and Anim Sci. (2021) 14:100216. 10.1016/j.vas.2021.100216 34825107 PMC8604666

[62] Roy BC Chowdhury SD Kabir SML. Effects of feeding Bacillus subtilis to heat stressed broiler chickens with or without an antibiotic growth promoter. Asian J Med Biol Res. (2015) 1:80–8. 10.3329/ajmbr.v1i1.25502

[63] Abudabos AM Aljumaah MR Alkhulaifi MM Alabdullatif A Suliman GM Sulaiman AR. Comparative effects of Bacillus subtilis and Bacillus licheniformis on live performance, blood metabolites and intestinal features in broiler inoculated with Salmonella infection during the finisher phase. Microb Pathog. (2020) 139:103870. 10.1016/j.micpath.2019.103870 31734387

[64] Al-owaimer AN Suliman GM Alyemni AH Abudabos AM. Effect of different probiotics on breast quality characteristics of broilers under Salmonella challenge. Italian J Anim Sci. (2014) 13:450–4. 10.4081/ijas.2014.3189

[65] Gil de los santos JR Storch OB Gil-turnes C. Bacillus cereus var toyoii and Saccharomyces boulardii increased feed efficiency in broilers infected with Salmonella enteritidis. Brit Poult Sci. (2005) 46:494–7. 10.1080/00071660500181461 16268108

[66] Knap I Lund B Kehlet AB Hofacre C Mathis G. *Bacillus* licheniformis prevents necrotic enteritis in broiler chickens. Avian Dis. (2010) 54:931–5. 10.1637/9106-101509-ResNote.1 20608542

[67] Knap I Kehlet AB Bennedsen M Mathis GF Hofacre CL Lumpkins BS. *Bacillus subtilis* (DSM17299) significantly reduces *Salmonella* in broilers. Poult Sci. (2011) 90:1690–4. 10.3382/ps.2010-01056 21753205

[68] Lee K Kyung D Lillehoj HS Jang SI Lee S. Immune modulation by *Bacillus* subtilis-based direct-fed microbials in commercial broiler chickens. Anim Feed Sci and Tech. (2015) 200:76–85. 10.1016/j.anifeedsci.2014.12.00628505587

[69] Lin Y Xu S Zeng D Ni X Zhou M Zeng Y. Disruption in the cecal microbiota of chickens challenged with Clostridium perfringens and other factors was alleviated by *Bacillus* licheniformis supplementation. PLoS ONE. (2017) 12:e0182426. 10.1371/journal.pone.0182426 28771569 PMC5542615

[70] Nusairat B McNaughton J Tyus J Wang J. Combination of xylanase and Bacillus direct-fed microbials, as an alternative to antibiotic growth promoters, improves live performance and gut health in sub-clinical challenged broilers. Int J Poult Sci. (2018) 17:362–6. 10.3923/ijps.2018.362.366

[71] Musa BB Duan Y Khawar H Sun Q Ren Z Mohamed MAE. Bacillus subtilis B21 and *Bacillus licheniformis* B26 improve intestinal health and performance of broiler chickens with *Clostridium perfringens*-induced necrotic enteritis. J Anim Physiol Anim Nutr. (2019) 103:1039–49. 10.1111/jpn.13082 31016810

[72] Aljumaah MR Alkhulaifi MM Abudabos AM Aljumaah RS Alsaleh AN Stanley D. *Bacillus* subtilis PB6 based probiotic supplementation plays a role in the recovery after the necrotic enteritis challenge. PLoS ONE. (2020) 15:e0232781. 10.1371/journal.pone.0232781 32555739 PMC7302482

[73] Ahmat M Cheng J Abbas Z Cheng Q Fan Z Ahmad B. Effects of *Bacillus* amyloliquefaciens LFB112 on growth performance, carcass traits, immune, and serum biochemical response in broiler chickens. Antibiotics. (2021) 10:1427. 10.3390/antibiotics10111427 34827365 PMC8614806

[74] Ogbuewu IP Okoro VM Mbajiorgu EF Mbajiorgu CA. Yeast (*Saccharomyces cerevisiae*) and its effect on production indices of livestock and poultry–a review. Comp Clin Pathol. (2019) 51:669–77. 10.1007/s00580-018-2862-7

[75] Hayat TA Sultan A Khan RU Khan S Hassan ZU Ullah R. Impact of organic acid on some liver and kidney function tests in Japanese Quails, Coturnix coturnix japonica. Pak J Zool. (2014) 46:1179–82.

[76] Lee K Lillehoj HS Jang SI Li G Lee S Lillehoj EP. Effect of Bacillus-based direct-fed microbials on Eimeria maxima infection in broiler chickens. Comparative Immunology, Microbiol and Infectious Dis. (2010) 33:e105–10. 10.1016/j.cimid.2010.06.001 20621358

[77] Bodinga BM Hayat KH Liu X Zhou J Yang X Ismaila A. Effects of *Bacillus subtilis* DSM 32315 on Immunity, nutrient transporters and functional diversity of cecal microbiome of broiler chickens in necrotic enteritis challenge. J World Poult Res. (2020) 10:527–44. 10.36380/jwpr.2020.61

[78] Santoso U Tanaka K Ohtani S. Effect of dried *Bacillus subtilis* culture on growth, body composition and hepatic lipogenic enzyme activity in female broiler chicks. Brit J Nutr. (1995) 74:523–9. 10.1079/BJN19950155 7577890

[79] Khajeh Bami M Afsharmanesh M Ebrahimnejad H. Effect of dietary *Bacillus* coagulans and different forms of zinc on performance, intestinal microbiota, carcass and meat quality of broiler chickens. Probiotics Antimicrob Proteins. (2020) 12:461–72. 10.1007/s12602-019-09558-1 31134523

[80] Berkes J Viswanathan V Savkovic S Hecht G. Intestinal epithelial responses to enteric pathogens: effects on the tight junction barrier, ion transport, and inflammation. Gut. (2003) 52:439–51. 10.1136/gut.52.3.439 12584232 PMC1773546

[81] Awad WA Hess C Hess M. Enteric pathogens and their toxin induced disruption of the intestinal barrier through alteration of tight junctions in chickens. Toxins. (2017) 9:E60. 10.3390/toxins9020060 28208612 PMC5331439

[82] Hernandez-Patlan D Solis-Cruz B Adhikari B Pontin KP Latorre JD Baxter MF. Evaluation of the antimicrobial and intestinal integrity properties of boric acid in broiler chickens infected with Salmonella enteritidis: proof of concept. Res Vet Sci. (2019) 123:7–13. 10.1016/j.rvsc.2018.12.004 30579139

[83] Abudabos AM Alyemni AH Dafalla YM Khan RU. Effect of organic acid blend and Bacillus subtilis alone or in combination on growth traits, blood biochemical and antioxidant status in broilers exposed to *Salmonella* typhimurium challenge during the starter phase. J Appl Anim Res. (2016) 45:538–42. Available online at: 10.1080/09712119.2016.1219665

[84] Abudabos AM Alhouri HAA Alhidary IA Nassan MA Swelum AA. Ameliorative effect of *Bacillus subtilis*, Saccharomyces boulardii, oregano, and calcium montmorillonite on growth, intestinal histology, and blood metabolites on *Salmonella*-infected broiler chicken. Environ Sci Pollut Res Int. (2019) 26:16274–8. 10.1007/s11356-019-05105-1 30977003

[85] Lei X Piao X Ru Y Zhang H Péron A Zhang H. Effect of *Bacillus* amyloliquefaciens-based direct-fed microbial on performance, nutrient utilization, intestinal morphology and caecal microflora in broiler chickens. Asian Austral J Anim Sci. (2015) 28:239–46. 10.5713/ajas.14.0330 25557820 PMC4283169

[86] Zhang B Zhang H Yu Y Zhang R Wu Y Yue M. Effects of *Bacillus* coagulans on growth performance, antioxidant capacity, immunity function, and gut health in broilers. Poult Sci. (2021) 100:101168. 10.1016/j.psj.2021.101168 33975039 PMC8131733

[87] Samany M Yamauchi K. Histological alterations of intestinal villi in chickens fed dried Bacillus subtilis var. natto. Comp Biochem Physiol A Mol Integr Physiol. (2002) 133:95–104. 10.1016/S1095-6433(02)00121-6 12160875

[88] Allen PC. Nitric oxide production during *Eimeria tenella* infections in chickens. Poult Sci. (1994) 76:810–3. 10.1093/ps/76.6.810 9181612

[89] Lillehoj HS Li GX. Nitric oxide production by macrophages stimulated with coccidian sporozoites, lipopolysaccharide, or interferon- γ and its dynamic changes in SC and TK strains of chickens infected with *Eimeria tenella*. Avian Dis. (2004) 48:244–53. 10.1637/7054 15283411

[90] Jang SI Jun MH Lillehoj HS Dalloul RA Kong IK Kim S. Anticoccidial effect of green tea-based diets against *Eimeria maxima*. Vet Parasitol. (2007) 144:172–5. 10.1016/j.vetpar.2006.09.005 17027157

[91] Konieczka P Sandvang D Kinsner M Szkopek D Szyrynska N Jankowski J. *Bacillus*-based probiotics affect gut barrier integrity in different ways in chickens subjected to optimal or challenge conditions. Vet Microbiol. (2022) 265:109323. 10.1016/j.vetmic.2021.109323 34974377

[92] Mora Z Macías-Rodríguez ME Arratia-Quijada J Gonzalez-Torres YS Nuño K Villarruel-López A. Clostridium perfringens as foodborne pathogen in broiler production: pathophysiology and potential strategies for controlling Necrotic enteritis. Animals. (2020) 10:1718. 10.3390/ani10091718 32972009 PMC7552638

[93] Belote BL Tujimoto-Silva A Hümmelgen PH Sanches AWD Wammes JCS Hayashi RM. Histological parameters to evaluate intestinal health on broilers challenged with Eimeria and Clostridium perfringens with or without enramycin as growth promoter. Poult Sci. (2018) 97:2287–94. 10.3382/ps/pey064 29660058

[94] Ogbuewu IP Emenalom OO Okoli IC. Alternative feedstuffs and their effects on blood chemistry and haematology of rabbits and chickens: a review. Comp Clin Pathol. (2015) 26:277–86. 10.1007/s00580-015-2210-0

[95] Lee KW Lillehoj HS Jang SI Lee SH Bautista DA Siragusa GR. Effect of *Bacillus* subtilis-based direct-fed microbials on immune status in broiler chickens raised on fresh or used litter. Asian Aust J Anim Sci. (2013) 26:1592–7. 10.5713/ajas.2013.13178 25049746 PMC4093815

[96] Abdel-Moneim AE Selim DA Basuony HA Sabic EM Saleh AA Ebeid TA. Effect of dietary supplementation of Bacillus subtilis spores on growth performance, oxidative status, and digestive enzyme activities in Japanese quail birds. Trop Anim Health Prod. (2020) 52:671–80. 10.1007/s11250-019-02055-1 31485898

[97] Zhang X Calvert RA Sutton BJ Dore KA. IgY: a key isotype in antibody evolution. Biol Rev Camb Philos Soc. (2017) 92:2144–56. 10.1111/brv.12325 28299878

[98] Balan P Sik-Han K Moughan PJ. Impact of oral immunoglobulins on animal health: a review. Anim Sci J. (2019) 90:1099–110. 10.1111/asj.13258 31270894

[99] Luan SJ Sun YB Wang Y Sa RN Zhang HF. *Bacillus* amyloliquefaciens spray improves the growth performance, immune status, and respiratory mucosal barrier in broiler chickens. Poult Sci. (2019) 98:1403–9. 10.3382/ps/pey478 30285152

[100] Wang Y Heng C Zhou X Cao G Jiang L Wang J. Supplemental *Bacillus subtilis* DSM 29784 and enzymes, alone or in combination, as alternatives for antibiotics to improve growth performance, digestive enzyme activity, anti-oxidative status, immune response and the intestinal barrier of broiler chickens. Brit J Nutr. (2021) 125:494–507. 10.1017/S0007114520002755 32693847 PMC7885174

[101] Rajput IR Li LY Xin X Wu BB Juan ZL Cui ZW. Effect of *Saccharomyces boulardii* and *Bacillus subtilis* B10 on intestinal ultrastructure modulation and mucosal immunity development mechanism in broiler chickens. Poult Sci. (2013) 92:956–65. 10.3382/ps.2012-02845 23472019

[102] Lalloo R Maharajh D Görgens J Gardiner N. A downstream process for production of a viable and stable Bacillus cereus aquaculture biological agent. Appl Microbiol Biotechnol. (2010) 86:499–508. 10.1007/s00253-009-2294-z 19921182

[103] Bhat AR Irorere VU Bartlett T Hill D Kedia G Morris MR. *Bacillus subtilis* natto: a non-toxic source of polyg-glutamic acid that could be used as a cryoprotectant for probiotic bacteria. AMB Express. (2013) 3:36. 10.1186/2191-0855-3-36 23829836 PMC3720193

[104] Markowiak P Slizewska K. The role of probiotics, prebiotics and synbiotics in animal nutrition. Gut Pathog. (2018) 10:21. 10.1186/s13099-018-0250-0 29930711 PMC5989473

